# Supplementary material for: The Expression of Adipogenic Marker Is Significantly Increased in Estrogen-Treated Lipedema Adipocytes Differentiated from Adipose Stem Cells In Vitro
Source: Biomedicines. 2024 May 9;12(5):1042. doi: 10.3390/biomedicines12051042 (PMC11117526; doi:10.3390/biomedicines12051042)
Supplement: Supplementary file 1 [file biomedicines-12-01042-s001.zip › biomedicines-2965329-supplementary.pdf]

Supplementary material

The Expression of Adipogenic Marker Is Significantly Increased in Estrogen-Treated Lipedema Adipocytes Differentiated from Adipose Stem Cells In Vitro

Sara Al-Ghadban [1,*], Spencer U. Isern [1], Karen L. Herbst [2] and Bruce A. Bunnell [1,*]

[1] Department of Microbiology, Immunology and Genetics, University of North Texas Health Science Center, Fort Worth, TX 76107, USA; spencer.isern@unthsc.edu
[2] Total Lipedema Care, Tucson, AZ 85715, USA; kaherbst@gmail.com
* Correspondence: sara.al-ghadban@unthsc.edu (S.A.-G.); bruce.bunnell@unthsc.edu (B.A.B.)

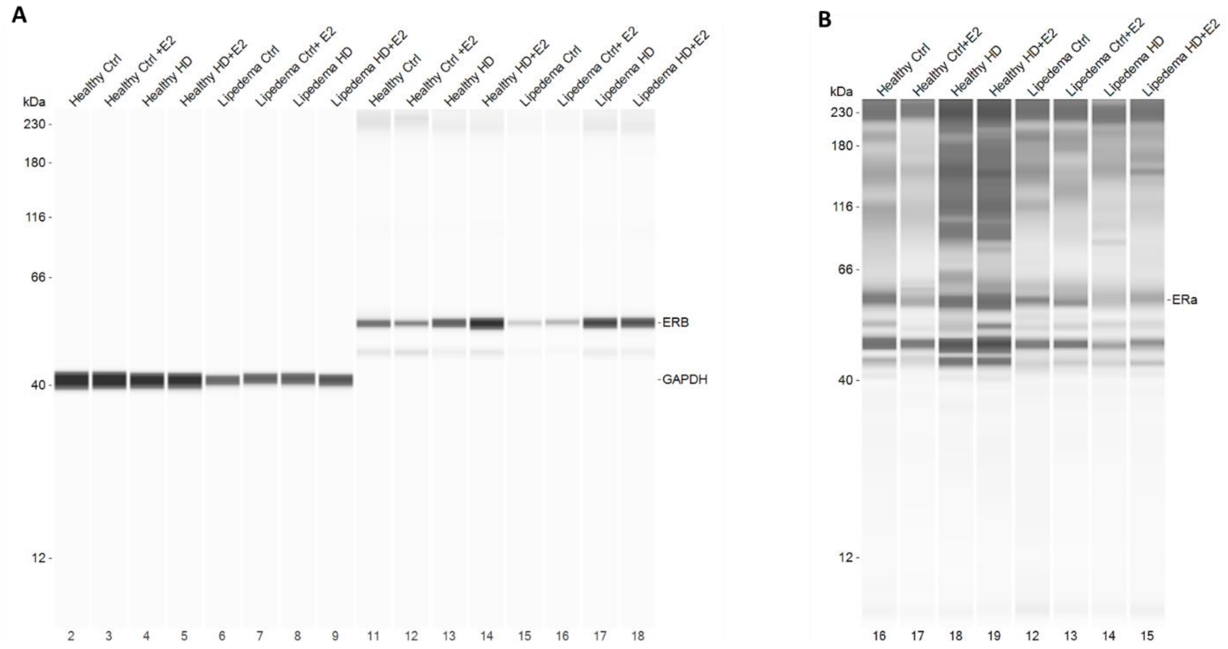

Figure S1. Western Blot showing the expression of (A) GAPDH (capillaries 2-9) and ERβ (capillaries 11-18) and (B) ERα in healthy and lipedema ASCs in 2D monolayer culture. The samples were run on a 12-230 kDa capillary kit.

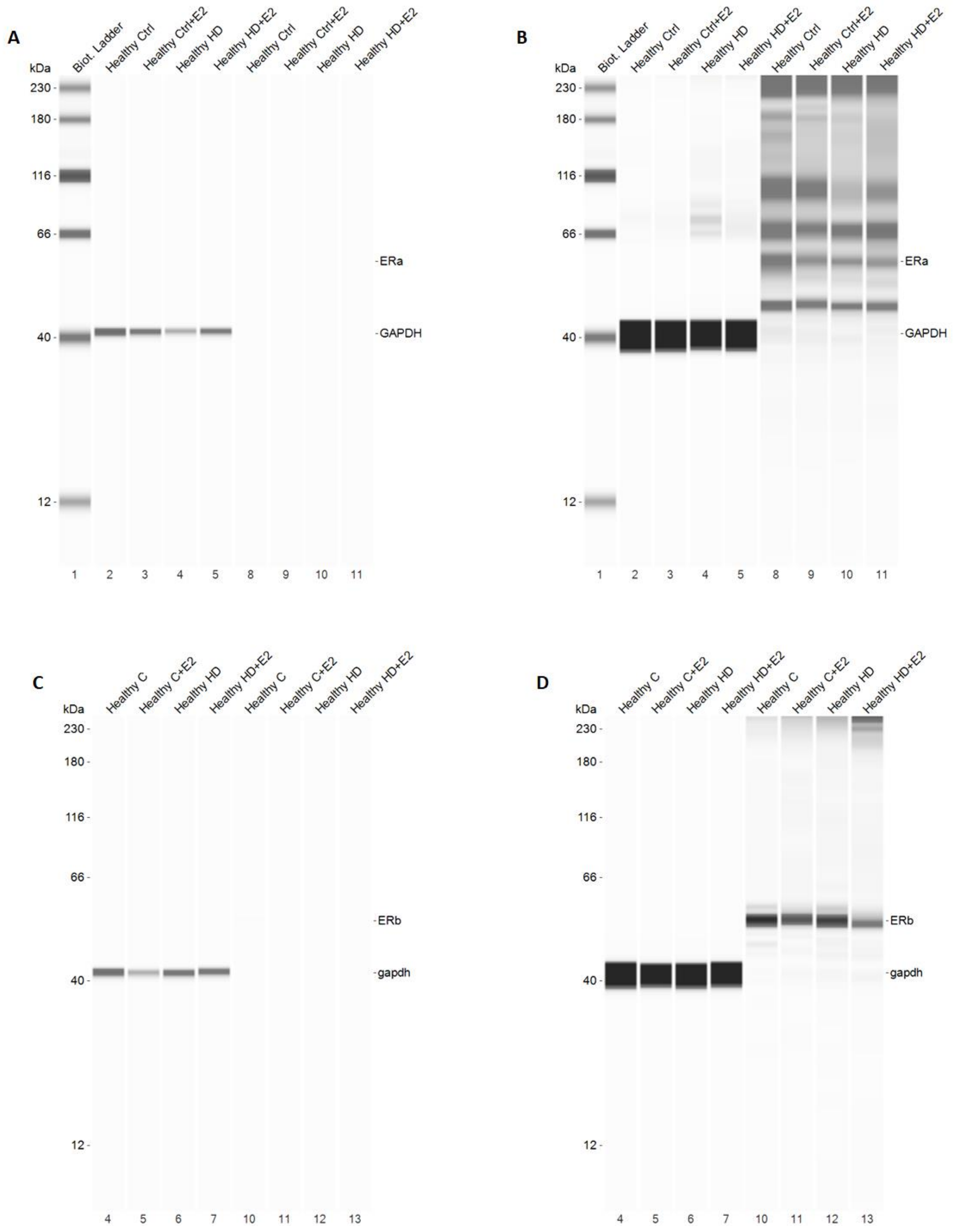

Figure S2. Western Blot showing the expression of (A, B) ERα and GAPDH and (C, D) ERβ and GAPDH in healthy spheroids. The samples were run on a 12-230 kDa capillary kit. A-B, and C-D are different exposures of the same run.

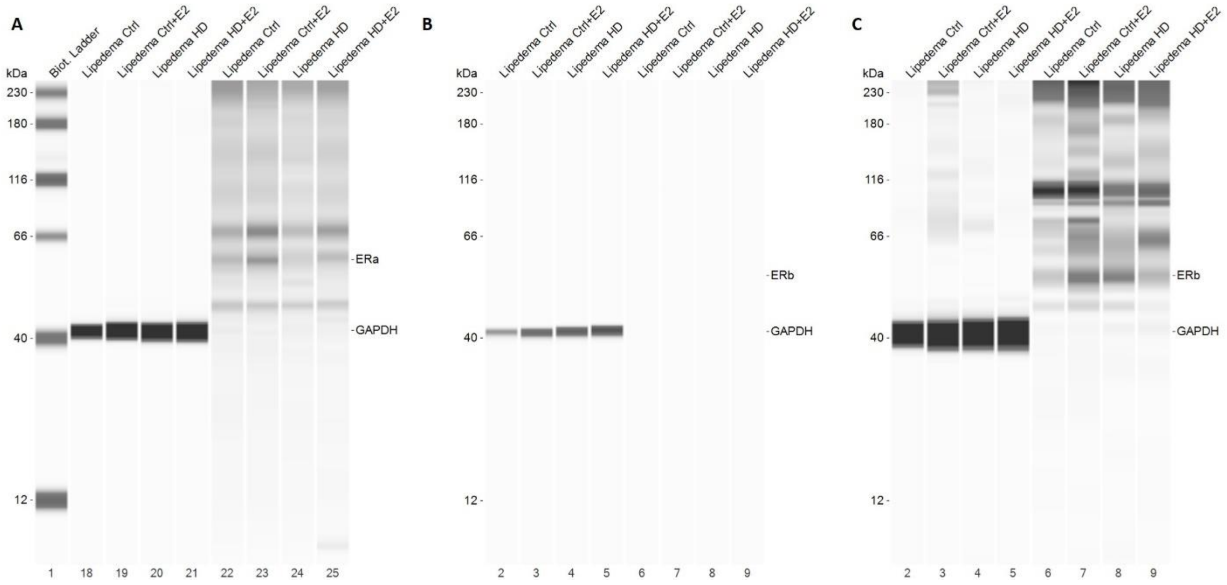

Figure S3. Western Blot showing the expression of (A) ERα and GAPDH and (B, C) ERβ and GAPDH in lipedema spheroids. The samples were run on a 12-230 kDa capillary kit. B,C are different exposures of the same run.

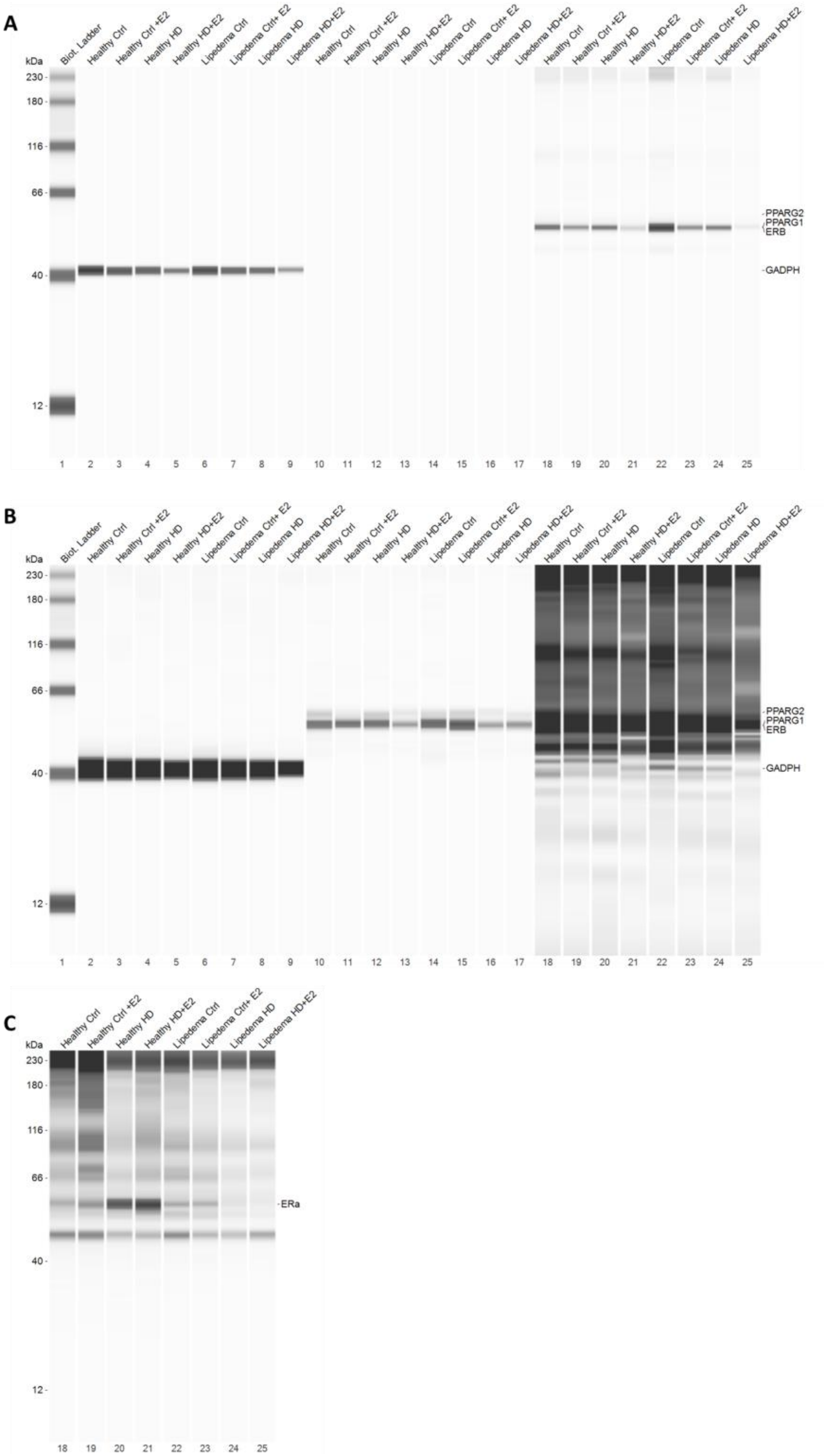

Figure S4. A, B. Western Blot showing the expression of GAPDH (capillaries 2-9), total PPARg (capillaries 10-17), ERβ (capillaries 18-25), and C. Western Blot showing the expression of ERα in healthy and lipedema adipocytes in 2D monolayer cultures. The samples were run on a 12-230 kDa capillary kit. A-B are different exposures of the same run.

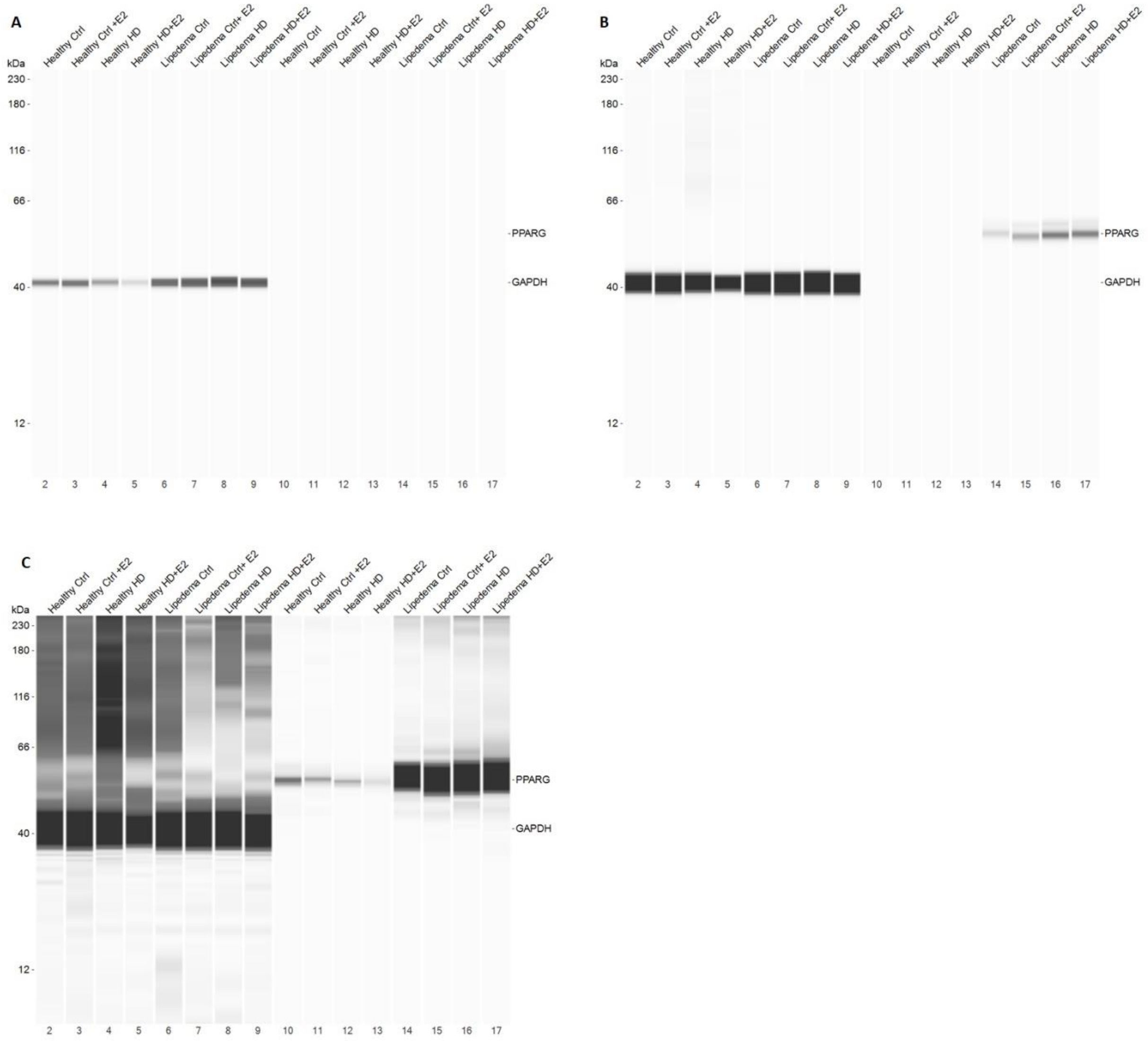

Figure S5. Western Blot showing the expression of GAPDH (capillaries 2-9), and total PPARg (capillaries 10-17) in adipogenic differentiated spheroids. The samples were run on a 12-230 kDa capillary kit. A-C are different exposures of the same run

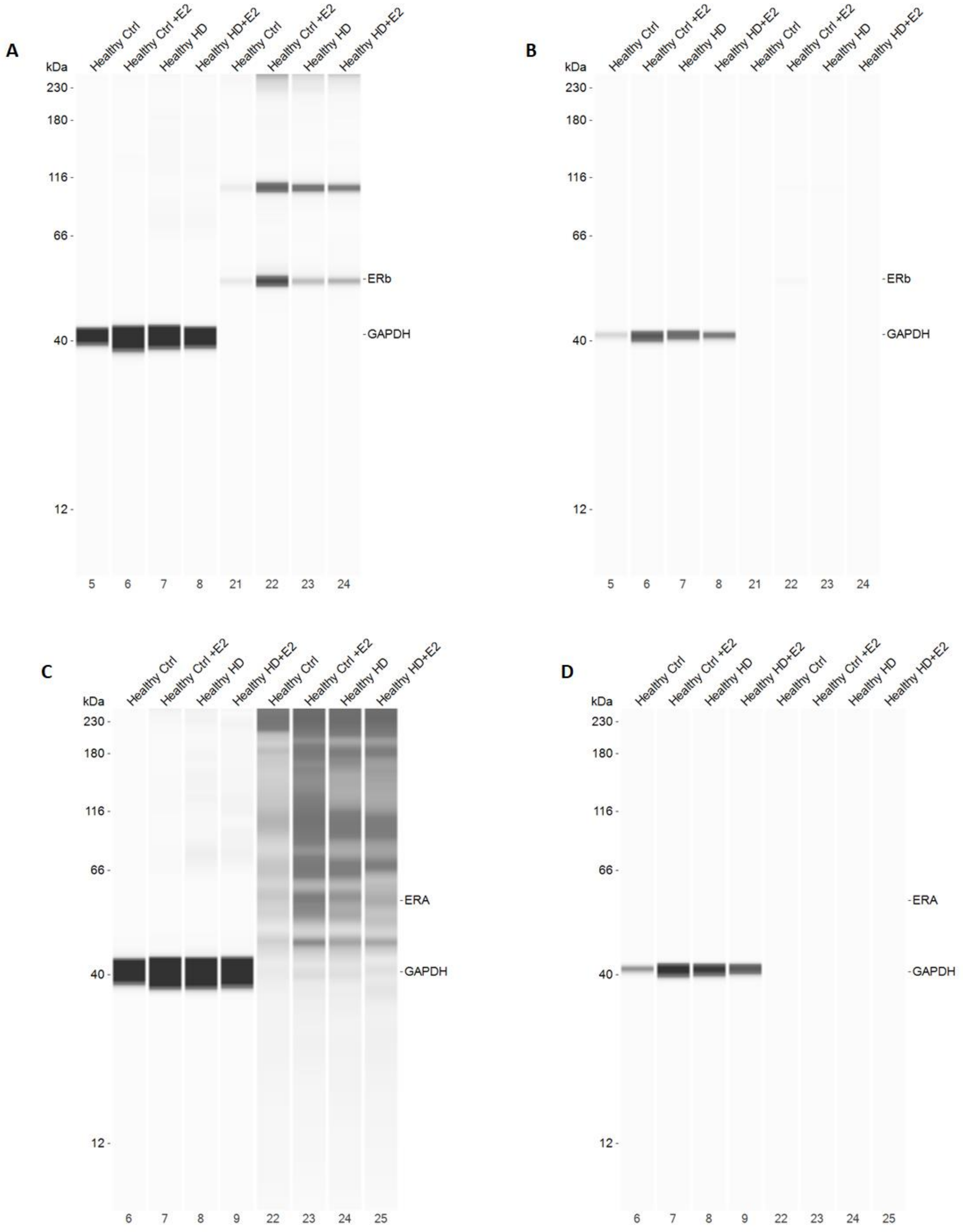

Figure S6. Western Blot showing the expression of (A, B) ERβ and GAPDH and (C, D) ERα and GAPDH in adipogenic differentiated healthy spheroids. The samples were run on a 12-230 kDa capillary kit. A-B, C-D are different exposures of the same run.

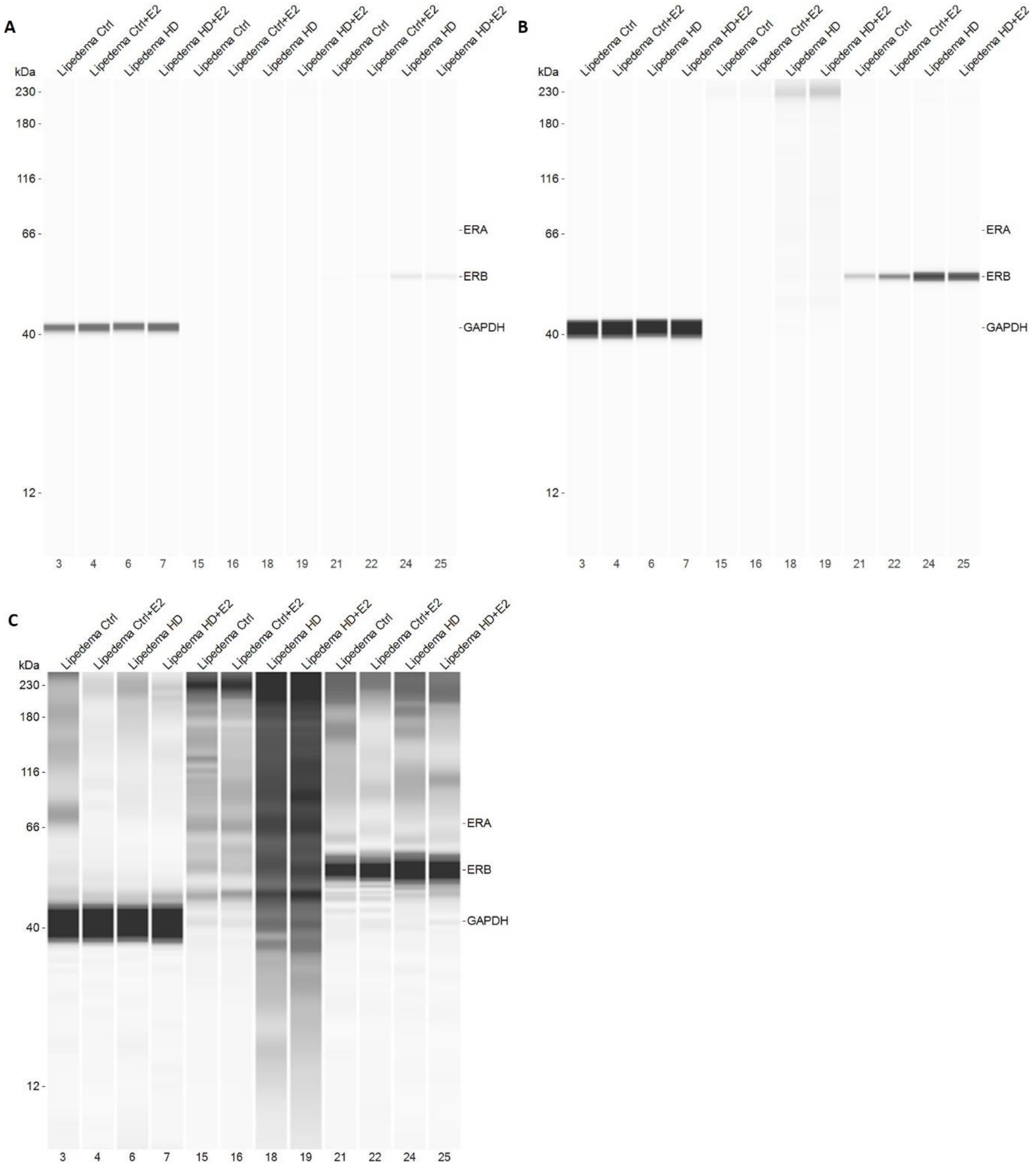

Figure S7. Western Blot showing the expression of GAPDH, ERα and ERβ in adipogenic differentiated lipedema spheroids. The samples were run on a 12-230 kDa capillary kit. A-C are different exposures of the same run.

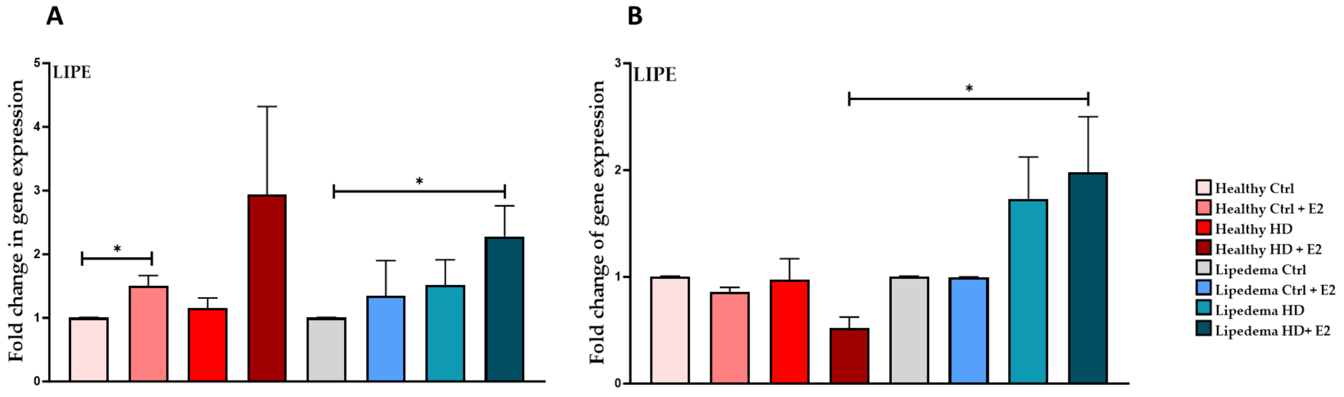

Figure S8. RT-PCR showed increased expression of LIPE in E2-HD lipedema treated (A) adipocytes in 2D culture and (B) differentiated spheroids compared to untreated control cells (n=3, *p<0.05).
